# Coverage of Emotion Recognition for Common Wearable Biosensors

**DOI:** 10.3390/bios8020030

**Published:** 2018-03-24

**Authors:** Terence K.L. Hui, R. Simon Sherratt

**Affiliations:** Department of Biomedical Engineering, School of Biological Sciences, The University of Reading, Reading RG6 6AY, UK; sherratt@ieee.org

**Keywords:** wearable biosensors, emotion recognition, emotion prediction, physiological specificity, basic emotions, PPG (photoplethysmography), EDA (electrodermal activity), EMG (electromyography), skin temperature, orienting response

## Abstract

The present research proposes a novel emotion recognition framework for the computer prediction of human emotions using common wearable biosensors. Emotional perception promotes specific patterns of biological responses in the human body, and this can be sensed and used to predict emotions using only biomedical measurements. Based on theoretical and empirical psychophysiological research, the foundation of autonomic specificity facilitates the establishment of a strong background for recognising human emotions using machine learning on physiological patterning. However, a systematic way of choosing the physiological data covering the elicited emotional responses for recognising the target emotions is not obvious. The current study demonstrates through experimental measurements the coverage of emotion recognition using common off-the-shelf wearable biosensors based on the synchronisation between audiovisual stimuli and the corresponding physiological responses. The work forms the basis of validating the hypothesis for emotional state recognition in the literature and presents coverage of the use of common wearable biosensors coupled with a novel preprocessing algorithm to demonstrate the practical prediction of the emotional states of wearers.

## 1. Introduction

Emotion has not been conclusively defined after more than a century of research [1,2], but the significance of the functions of emotion are less arguable than its definition [3,4,5]. The promotion of emotional responses not only can balance our physical health through the nervous and endocrine systems to reach the goal of homoeostasis [6], recent research on neuroimaging also proves that our everyday behaviour is a close integration of both cognition and emotion [7,8]. Moreover, human beings located in different contexts (or environments) [9], having different experiences [10], having physiological impairments due to health [11] or ageing [12] or having mental illness [13] may experience abnormal emotional behaviours when the same stimuli are applied. Consequently, emotion recognition may become a personalised service according to individual’s spatial and temporal situations [14,15,16,17,18]. A major problem in common emotion recognition system lies in a successful elicitation of target emotions for further processing since the perceived emotions may not be the induced emotions [19,20].

The measurement of human emotions is a crucial step in Artificial Intelligence (AI) to enable computers to have affective communication with humans [21,22]. Ubiquitous computing using the Internet of Things (IoT) has implemented AI technologies into smart homes and smart cities [23], since it is forced to interact with human users by sensing, recognising and predicting their intentions based on the prevailing phenomenon of disappearing user interfaces (DUIs) [24]. Emotional responses are best captured by a DUI through natural and intuitive interactions since human perception of emotions may consist of both conscious and unconscious processes [25,26]. Remote and on-body sensing are both good DUI candidates for individual emotion recognition, but remote sensing such as visual data capturing or radio frequency leveraging is still application limited, especially in ambulatory activities; thus, wearable sensors may be a better choice in pervasive sensing applications. However, wearable “things” in IoT normally face challenges such as limited processing power, size constraints and limited battery capacities. Therefore, a meticulous selection of embedded sensors with a lightweight methodology for emotion recognition is a basic requirement in designing affective wearables in the IoT environment and forms the focus of this paper.

Section 2 presents the related work done by previous research. The experiments, tools and the framework design based on DUI for the current research are depicted in Section 3. This is followed by the result of the experiments in Section 4. Further discussion in Section 5 analyses the validation of emotion elicitation and the coverage of emotion recognition for each individual and combined wearable sensors. In the last section, we conclude our findings and lay down the groundwork for future research.

## 2. Related Work

**Figure 1:**
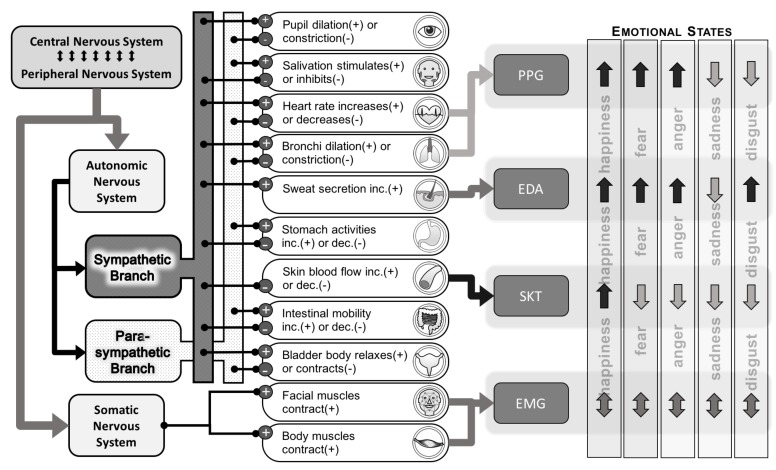
Simplified emotion recognition using common wearable biosensors. SKT, skin temperature.

Emotions can be recognised by mapping specific patterns of the measured biosignals based on physiological responses promoted by an emotional perception [27,28]. Many researchers have produced empirical evidence showing that the human brain orchestrates the autonomic nervous system (ANS) and endocrine systems, which then innervate the visceral organs to produce autonomic specificity [29,30,31]. A comprehensive study of an affective wearable illustrated by Picad [32] set off the application in wearable physiological measurements on emotion recognition, which is a sub-branch of affective computing [21]. During the past two decades, research on emotion wearables has emerged aiming to enable ambulatory emotion recognition by incorporating the advancement in sensing technologies [33,34], networking technologies [35,36] and emotion recognition methodologies [37,38]. Research on emotion recognition for wearables has focused mainly on using common biomedical sensors, and the collection of biosignals as a training dataset is fed to a classifier based on modern machine learning algorithms. Recognition accuracy varies according to the choice of sensor signals and their derivatives, the placement of sensors, the presentation and types of stimuli, as well as the different classification algorithms [39,40,41]. Recent research indicates an upward trend in the number of features extracted from those physiological signals promoted by emotion perception for boosting the prediction accuracy based on supervised learning algorithms, and that number can even exceed 100 in certain research methodologies [42,43,44]. Principle component analysis (PCA) may help reduce the number of effective variables, but the stable control of a high number of features is still a significant challenge particularly in wearable devices [45,46]. However, original research on autonomic specificity [27,28,29,47,48,49,50] did illustrate a simple relationship between some basic discrete emotional states and the associated physiological responses such as the variations in heart rate, the magnitude of changes in skin conductance, the subtle fluctuations in fingertip temperature and also the relatively complicated contractions on facial and skeletal muscles. Figure 1 depicts a simplified relationship between the nervous systems, some of the affected visceral organs, common wearable biosensors for emotional state recognition and the ANS specificity that were demonstrated for some discrete emotions from previous empirical studies [29]. The figure only shows those autonomic patterns about which most researchers agree, and the emotion recognition process of the current study also relies on this pattern. Motor programs activating the skeletal muscles of the face and certain parts of the body are also believed to be concomitant to the ANS during emotional responses and are normally treated as one of the contributors to the physiological specificity for emotions [51,52,53].

Collecting the correct moment of physiological data for feeding a well-trained statistical prediction machine learning algorithm is equally important in affective application, especially in the personalised smart services provided by IoT environments where an identification of affective moment should initiate certain operations. Orienting response (OR) has been heavily researched as an indication of attention to environmental change for animals and humans [54,55], and the OR hypothesis is further strengthened by the P3 component of the event-related potential in neuroscience research based on previous electroencephalography (EEG) measurements [56,57]. Cardiac deceleration and skin conductance changes are common indications for initiating the OR process when novel audiovisual stimuli are applied to humans, activating emotional perceptions. Recent OR research also hypothesises that the variations of cardiac deceleration can distinguish between pleasant and unpleasant emotions through audiovisual cues [58,59,60]. A synchronisation between audiovisual stimuli and the concomitant physiological variables may be able to capture this OR process, which facilitates the validation of a successful emotion elicitation for further analysis and operations. According to Kreibig’s review [29], there is no standard for the averaging interval of the physiological measurement (varying from 0.5 s–300 s and most often being 30 s and 60 s), which is the temporal quality of the elicited emotions. Therefore, the correct moment of the measurement interval from start to finish defines whether the details of the emotional responses are captured.

There has been much research about emotion recognition based on physiological patterning, such as those using machine learning, as previously discussed and that will not be explored in this paper. The current research focuses on the preprocessing of emotion recognition, particularly on the analysis of the validation of successful affective moments stimulated by audiovisual cues, and the coverage of common wearable biosensors.

## 3. Materials and Methods

The experimental protocol for this study was approved by the Research Ethics Committee of the University of Reading #SBS17-18 10. Five male and five female adults with an average age of 44.9 years (STD = 10.7) without any diagnosed medical conditions (e.g., diabetes, heart disease, sweating disorders such as hyperhidrosis or hypohidrosis) were chosen to participate in the study. Each participant was presented with several types of emotional stimuli, and their corresponding physiological responses were recorded using the four types of non-invasive wearable biosensors mentioned in this paper. Two out of the ten subjects, a male and a female, were invited to extend the testing for a longer version of audiovisual stimuli by watching a TV program eliciting “joy” emotions in order to demonstrate real-time continuous emotion recognition using the current framework.

### 3.1. emotionWear Framework

**Figure 2:**
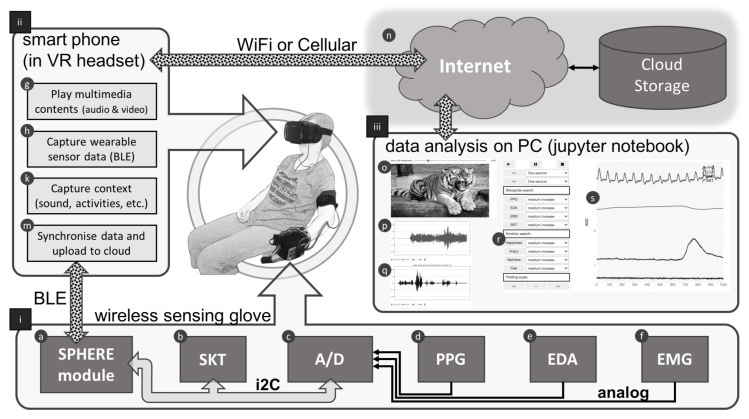
Block diagram of emotionWear emotion recognition framework. SPHERE, Sensor Platform for HEalthcare in a Residential Environment.

A framework was built for the current research as a complete emotion recognition system from stimulation of emotions to registration of the corresponding physiological responses. Synchronisation between stimuli and responses is established for easy monitoring and referencing by using an Android smartphone acting as a central control centre. Figure 2 depicts a block diagram of the whole emotionWear framework, which consists of the following components (the references (i), (ii), (iii), ⓐ, ⓑ, ⓒ, etc., in the descriptions below refer to the labels in the drawing):

(i) Wearable Sensing Glove
  Biomedical sensors (PPG, photoplethysmogram; EDA, electrodermal activity; SKT, fingertip temperature; EMG, electromyography) are mounted on a sensing glove made from a wrist support with Velcro straps for easy installation and setup. The PPG and EDA sensors are attached to the inside of the glove touching the thenar region of the hand of a subject; the SKT digital temperature sensor is attached to the tip of a middle finger; and the EMG sensor is attached to the triceps area of the same arm. All these sensors are wired to a SPHERE (Sensor Platform for HEalthcare in a Residential Environment) (http://www.irc-sphere.ac.uk) module ⓐ, which is a wireless wearable platform having built-in gyroscopes and accelerometer for activity recognition and a wireless connectivity using Bluetooth Smart [61]. PPG ⓓ, EDA ⓔ and EMG ⓕ sensors produce analogue outputs and are connected to a 4-channel A/D (analogue to digital) converter ⓒ allowing the SPHERE module to sample the readings at 100 Hz. The SKT sensor ⓑ, due to the small variation (less than 1 degree centigrade), is connected directly to the SPHERE module through I2C (Inter-Integrated Circuit) bus and sampled at the same rate. Data sampling at 100 Hz is sent as a notification package of 20 bytes in length including all 4 sensor data readings when the SPHERE module is connected and subscribed by a BLE (Bluetooth Low Energy) client, which, in this case, is an Android smartphone. All wearable sensors used in the emotionWear sensing glove are common off-the-shelf biomedical sensors, and the details of the sensors are listed in Appendix A.
(ii) Android smartphone:
  An Android smartphone (model: Google Pixel with Android OS Version 7.1.2) with tailor-made application software acts as a central control in the emotionWear framework. The application software is comprised of four different modules: ⓖ allows manual selection of multimedia contents previously downloaded to a particular folder as separate files and displayed as two images on the smartphone’s screen such that when wearing a VR headset, the user sees a 2-dimensional image presented to each eye. Audio content is applied through an earphone; therefore, the subject can be treated as being isolated from the outside world and focussed only on the multimedia contents. ⓗ During the multimedia playback, the corresponding signals measured by the wearable biosensors in (i) are collected through the BLE connection and saved to a file. ⓚ The ambient sound is kept to a minimum during the study, and audio from the surroundings is picked up by the smartphone’s built-in microphone to record any sound generated by the subject during the test. ⓜ Once a study session is over, the smartphone pushes the related data files (i.e., biosignals and the context) to the Internet through WiFi or cellular connection and stored in the cloud ⓝ.
(iii) Data analysis
  Data analysis is done using interactive Python (iPython), based on the jupyter notebook application (http://www.jupyter.org), which has an active community providing third-party libraries for data sciences [62]. The iPython code is written to show a dashboard view of the emotionWear framework for analysing the synchronised stimulation (i.e., the multimedia contents including visual and audio) and the accompanying physiological responses (i.e., the signals picked up by the 4 wearable biosensors, together with the capture of the environmental sound) under emotional perceptions. The visual stimulus is displayed as still images, which are the screen shots of the video in 100-ms intervals ⓞ; the audio part of the video is displayed as an amplitude envelope plot ⓟ; the capture of the environmental sound generated from the subject during the study is also plotted in the same scale ⓠ; a set of interactive control widgets of ipywidgets (https://ipywidgets.readthedocs.io/en/stable/) is implemented to move the dashboard view to the specific time instances according to the following criteria:
  - Slider: slide to anywhere on the time scale in the 100-ms interval, and the corresponding stimulus (video screen shot and audio segments) and responses (waveforms of the biosignals) at the selected time instance are displayed.
  - Threshold level detection for the four biosignals activates the comparison logic for small, medium and high threshold amplitudes preset by the program. Once the comparison output is positive, the slider is moved to the time instance of the comparison.
  - Threshold level detection for ANS specificity activates the comparison logic for ANS patterns comprising the four biosignals preset by the program. Once the comparison output is positive, the slider is moved to the time instance of the comparison.
  The actual waveforms for the corresponding biosignals in the time and frequency domains are displayed in ⓣ. These waveforms show the biosignals being sampled at 100 Hz synchronised with the multimedia stimuli in ⓞ ⓟ and the sound capture ⓠ.

### 3.2. Biosensors and Features Selection

Innervations happen to many human organs during an emotional physiological response (Figure 1 shows some examples), but non-invasive measurements cannot be conveniently applied to most of them. Researchers normally focus on the following four types of wearable biosensors due to them being (i) non-invasive, (ii) easily available and (iii) mature technologies for manufacturing and analysing, and we have also chosen them for the present study. Additionally, the feature extraction process requires special consideration according to the nature of emotional stimuli.

(a) PPG, EDA and SKT:
  PPG captures the blood volume pulses, which can then be used to derive features from both the time and frequency domains. EDA captures the skin conductance, and both the mean and standard deviation are extracted as features. Similar to EDA, SKT captures fingertip temperature with features derived from both the mean and standard deviation. Table 1 lists all features with the associated calculations.
  All features are calculated based on different rolling windows defined by the “window” parameters, which affect the resolution for temporal analysis. A short window such as 10 s is applied for most of the time domain features, but it is not reliable for frequency domain features, especially low frequency (LF) (0.04 Hz–0.15 Hz) [63]. While there are obvious variations in the high frequency (HF) features, the LF features are not stable since a frequency transform on low frequency components is not reliable due to insufficient data over a short time frame, e.g., a period of 25 s is required for one cycle of a 0.04-Hz signal in LF. For normal film watching, as well as real-time situations, emotional scenes are usually very short compared to the 5-min rule in medical cardiac evaluation [64,65].
(b) EMG:
  EMG indicates the degree of muscle contraction. Ekman has demonstrated that facial muscle contraction is concomitant to ANS specificity such that a motor program is initiated and generates specific facial expression [66]. Other than facial muscles, skeletal muscles of body parts are also studied by different researchers showing a system of emotional muscle response termed BCAS (body action coding system) [67,68]. However, there is not much empirical research supporting this system on emotion recognition. Facial expression detection may not be easily applied to a wearable sensor, so EMG in this study has been placed on forearm, biceps and triceps, but no significant relationship between these signals on those areas could be found. Finally, the EMG data were discarded in the current study.

### 3.3. Stimulation of Emotions

Visual cues are common and successful stimuli for emotion elicitation. Still images and film clips are traditional visual and audiovisual cues used in academic research to elicit specific emotions with standardised materials provided by different academic institutions for easy referencing, for example International Affective Picture System (IAPS) [69,70,71] from University of Florida and emotional film clips proposed by Gross and Levenson [72] and Schaefer et al. [73]. However, the effectiveness of these two types of visual stimuli for emotion elicitation is still debatable [74]. Still images seem to be less complex for investigation of single affective states [70], but emotional films may be higher in ecological validity [72]. Therefore, both still images and film clips are adopted in the current study. Selected pictures from IAPS are compiled as a slide show with 6 s displaying the picture and 20 s blank screen between pictures, and the selection criterion is based on the highest and lowest valence from the accompanying test report inside the IAPS package (file “AllSubjects_1-20” inside folder “IAPS Tech Report”). These slide shows are stored as film clips in mpeg layer-4 format (.mp4) without auditory content. Film clips [72,73] with the highest ranking or hit-rate were used as normal mp4 multimedia files with both visual and auditory contents. Converting picture slide shows into film clips allows both types of visual stimuli to share the same functions in the emotionWear framework. Table 2 lists the pictures (identified by the individual reference numbers in IAPS) and the film clips used to elicit the five basic emotions under study.

A longer film, around 30 min, was chosen as the audiovisual stimuli for continuous elicitation as an extension of the test for real-time emotion recognition. A commercial TV drama program “Friends, season 2, episode 1 from NBC” (https://www.youtube.com/watch?v=ydQ26w2TVEE&list=ELVOi8oFxJzT0) is targeted for “joy”, and this film has several moments of “joy” emotions.

### 3.4. Procedures

Each subject is briefed on the project and experiment by the investigator explaining the details listed on the “Information Sheet for Participants”. The investigator completes the questionnaire and collects basic details of participants (such as age range, sex, etc.) according to the “Questionnaire” form. The investigator also explains the potential risks of emotional harm from watching irritating materials especially those eliciting fear, disgust and anger emotions. Participants can refuse to watch those materials if they feel uncomfortable, thus the investigator will only select and show to the participants the appropriate visual stimuli. The investigator helps to choose one film clip from each emotion category for the study and the IAPS slide show under the participant’s agreement. The maximum duration for the film clips is less than 6 min, and the slide show is 9 min, plus the handling time between switching media, and the total study period is less than 1 h. The subject wears a VR headset with an Android smartphone installed inside the headset to show the emotional films and collect data from the wearable sensors through Bluetooth wireless connection. The subject may need to adjust the focus on the VR headset in order to get a clear picture according to the instructions from the investigator. The investigator will help the subject to wear a glove with the wearable sensors installed, and the sensors are wirelessly connected and controlled by the Android smartphone in the VR headset. The investigator will need to make sure all wearable sensors are properly installed and the biomedical signals picked up are at the highest quality. Three minutes are reserved to allow the subject to relax and at the same time, the baseline signals are collected to adjust the experimental data. The experiment will then start after the relax period, and the film watching and data collection are performed automatically. When the experiment stops, the investigator will help the subject to remove the sensors and the VR headset. The investigator will continue filling in the unfinished questionnaire and collect subjective feelings from the subject based on the Self-Assessment Manikin (SAM) form from the IAPS package. The experiment ends, and the subject can collect the “Information Sheet for Participants”, which shows the contact information if required for future communication.

## 4. Results

After a successful elicitation of emotions, the perception promotes physiological responses, which enable wearable sensors to perform biomedical measurements if the variations of the corresponding biosignals are within the limits of contemporary sensing technologies. Machine learning enables statistical prediction of emotions based on supervised learning of features extracted from a large database of physiological data with ground-truth labels according to previous research [42,43,44]. Our result applies autonomic specificity using the minimum number of biological variables for emotion recognition through simple feature comparison proposed by previous empirical studies [28,29,50]. The method can be effortlessly used in other areas requiring emotion recognition as an integral part of the whole system, especially those resource-limited smart sensors used in the IoT world. Three types of common biosensors (since EMG was discarded) that can be easily purchased are selected for the current research, so our result is repeatable with contemporary technology. The following subsections present the response-stimulus synchronisation (RSS) results of the emotionWear framework using the three different types of audiovisual stimuli dedicated to specific purposes:

(a) Still pictures:
  This visual stimulus contained 20 still pictures without auditory content, thus a relatively simple attention to the perception process was established. The pictures were separated into two groups with similar valence ratings within each group, but high valence contrast between the groups. RSS results showed the responses of all participants during the viewing of individual pictures for high and low valence, as well as the switching of pictures within-groups and between-groups.
(b) Short film clips:
  Film clips with dedicated emotional rankings and short durations (see Table 2) stimulated specific perceived emotions in participants, and the purposes of using RSS were (i) validation of emotion elicitation, (ii) comparison between perceived and felt emotions and (iii) pattern matching of biosignals with ANS specificity for successful and unsuccessful emotion elicitations.
(c) Longer version film clip:
  A longer film clip around 30 min with multiple emotional stimulation moments of ‘joy’ caught viewers’ attention and elicited corresponding emotions, and the RSS demonstrated the whole stimulation, attention, perception and autonomic responses process. Additionally, synchronisation was extended to the environmental context by demonstrating how the collected background sound was synchronised with the physiological responses during the emotional perceptions.

### 4.1. Still Pictures

**Figure 3:**
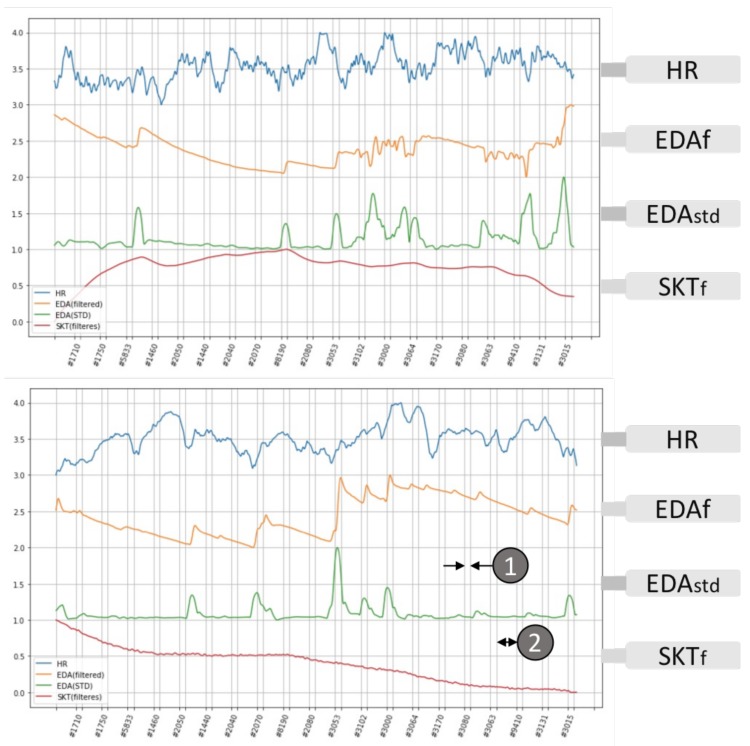
Physiological responses for still picture stimuli (IAPS). Each study session of emotion recognition using IAPS stimuli lasts a total of 520 s; the 20 chosen pictures in two categories listed in Table 2 are shown as still images on the display in sequence of six seconds per image ①, and the gaps between images are each filled with a 20-s black screen ②. The x-axis shows the arrangement of the two categories of IAPS pictures according to their unique reference numbers. All participants showed unpleasant emotion when the first negative valence picture was displayed (#3053). The upper picture shows a higher heart rate deceleration during the switching of emotional valence, and the lower picture depicts a more significant skin conductance change.

Twenty still pictures were chosen from the IAPS’s database of 1182 photos; the first group of 10 was selected with the highest valence rating and the second group of 10 for having the lowest values; thus, a maximum contrast in emotional valence was established between the two sets of picture stimuli (the ratings are found from the IAPS technical report [71] inside the IAPS package). The selected pictures were displayed in sequence according to Section 3; half of them were rated as high valence, which normally stimulate positive emotions such as happy, content or pleased; another half with a low rating should produce negative emotions such as distress, sadness or frustration. Each still picture was displayed for 6 s and was followed by a blank screen of 20 s; thus, a total of 20 × (20 s + 6 s) = 520 s was spent to complete one experiment for a sequence of 20 images. All subjects (100%) experienced an unpleasant emotion after switching to the negative valence picture group, and they all expressed the “disgust” emotion during the subjective feeling survey. Figure 3 shows two typical physiological responses from the wearable biosensors of the subjects watching the two categories of still pictures in sequence according to the timing shown in the horizontal axis.

A significant increase in EDA response was found during the switching from positive to extreme negative valence stimulus when the unpleasant picture group was displayed, as well as a moderate increase in the average heart rate, and simultaneously the fingertip temperature started to drop until the end of the study. Picture #3053 is the first unpleasant picture in the sequence, and all changes started from that moment. A significant change in skin conductance and a heart rate deceleration occurred at picture #3053 and also other moments switching to new pictures. However, not all of the switching of pictures, especially the high emotional valence pictures, caused the hypothesised OR activities, which illustrated the different initiation of the orienting response process, and the individual perception differences were demonstrated among the 10 subjects. A combination of an average increase in heart rate and skin conductance, together with a decrease in fingertip temperature agrees with those reported by Kreibig [29] for the basic emotion of “disgust (mutilation)”.

Previous research has shown that EDA seems to be the only reliable and consistent phasic OR indicator [57]. If the focus is diverted to EDA only, then pleasant emotional pictures (i.e., cute animals and babies) give less response, but unpleasant pictures stimulate more emotional perceptions. Additionally, the OR occurrences for subsequent unpleasant emotions keep reoccurring with increased delay from the emotional scenes after the initial response due to the audiovisual stimulations. The after study survey shows that the participants felt neutral to most of the selected high valence pictures, but perceived “disgust” and “fear” emotional states during the viewing of those low valence pictures.

### 4.2. Short Film Clips

Film clips used in this study were rated and classified by Schaefer et al. [73], and some films were also investigated by Gross and Levenson [72]. All film clips were downloaded from the related web site (http://www.ipsp.ucl.ac.be/FilmStim/) mentioned in the article. Section 3 describes the 15 film clips in five categories that have been used as audiovisual stimuli for eliciting the five discrete emotions under the current study, and the results are summarised in Table 3.

The 10 subjects were allowed to choose the type of emotional film clips since some of them were uncomfortable with certain types of emotional films, especially those with scary and disgusting scenes. No question was asked to identify repeated exposure to films they had previously watched, since this was part of the current study. Finally, a total of 48 film clips was used for this study, and only half of them could successfully elicit emotions. Table 4 shows the reasons for unsuccessful elicitation from the after study survey with the subjects when they could express their subjective feeling of the film clips they had watched. The most common reason seems to be insufficient watching time to initiate a perception, particularly those emotions related to “anger” and “sadness”. The next common reason is that the subjects could not feel the target emotions or even any emotion at all; this happened in all categories, but “anger” is the worst, with zero subjective elicitation. Repetitive exposure seems not as bad as we have anticipated since almost half of the films have been watched by the participants, but most of them could still perceive the target emotions, especially those related to “fear” and “disgust”.

Table 3 also shows that subjective feeling agrees with the target emotions on “joy” since the scenes are easily identifiable, although the subjects may not perceive the same emotion for most of the “joy” film clips. No subject agrees on “anger” emotion, and this matches with previous research that anger emotion is hard to elicit in film watching [75]. “Fear” and “disgust” are hard to distinguish for most of the subjects, and this phenomenon matches with previous studies indicating that disgust sometimes comes with “fear” emotion. The percentage of subjective feeling of emotion elicitation and the actual elicitation by measurement is also listed in the table, which reveals that there is no emotional perception even if the subjects have claimed the target responses of the film clips, especially those with low arousal levels.

**Figure 4:**
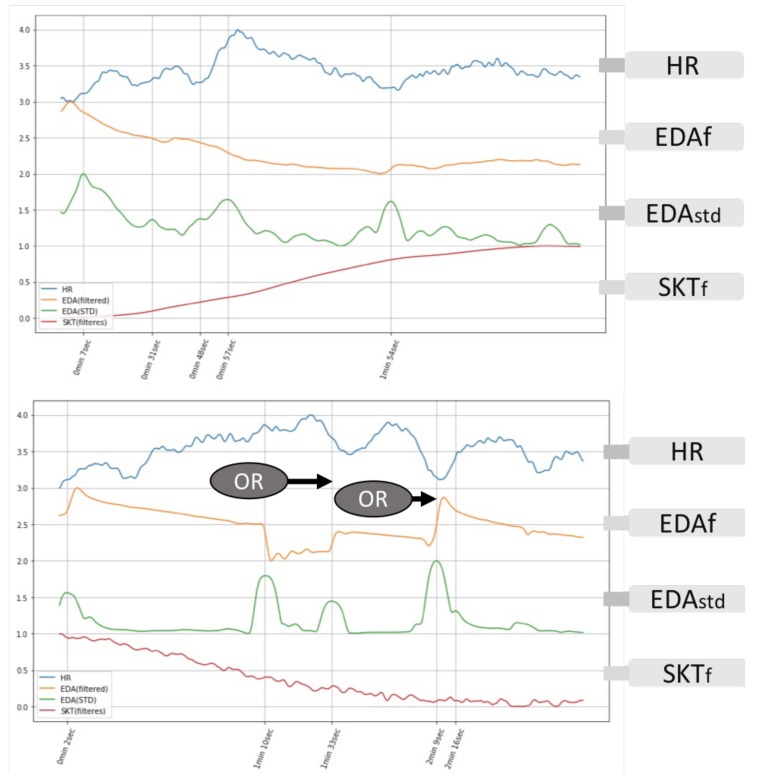
Orienting responses on unsuccessful (upper) and successful (lower) emotion elicitation of film clips.

OR analysis was used to validate the perception of the emotional stimuli by checking the HR deceleration for two seconds starting one second before the change of EDA response, and we have achieved an 85% accuracy for predicting a valid emotional perception. Figure 4 depicts typical waveforms showing the HR and EDA responses for successful and unsuccessful emotion elicitation.

After validating a successful emotion elicitation, the average levels for the three biosignals (HR, EDA and SKT) after an occurrence of OR were calculated and compared with the ANS specificity patterns (Figure 1), and we obtained a reasonable accuracy of emotion prediction. Table 5 shows the accuracy confidence of emotion recognition before and after the moment of OR (i.e., the assumption of successful emotion elicitation). The calculation was based on a 60-s interval after the OR moment for a successful emotion elicitation, and the other one was calculated using the whole film duration and segmented into 60-s intervals, then taking the average. All three variables showed a significant increase in the confidence level representing by the *p*-value, and the results of EDA and SKT met the criterion for statistical significance (*p* < 0.05). The HR feature is a more complicated variable affected simultaneously by both branches of the ANS, so a more sophisticated pattern must be investigated to increase the recognition accuracy. Validating the emotion elicitation moment seems to be critical in choosing the averaging interval for recognition.

### 4.3. Longer Version Film Clip

**Figure 5:**
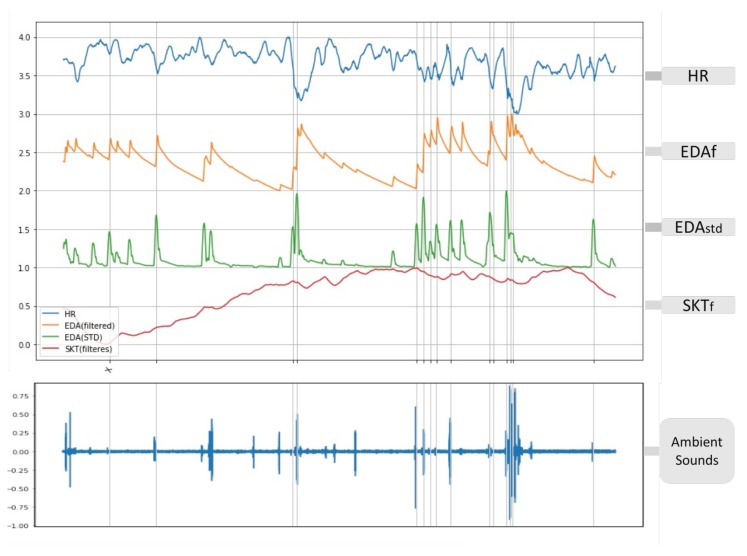
Physiological responses for continuous emotion elicitations.

A longer film (a TV program around 30 min) was used to extend the existing test to analyse the physiological responses captured by the current emotion recognition framework. The result verifies the detection of OR activities as validation of successful emotion elicitation and reviews the effectiveness of the recognition method using a comparison with ANS specificity patterns (average high in HR, EDA and SKT). A “joy” emotional film (TV program: Friends) having multiple and continuous emotion elicitation moments has elicited “joy” emotion with high arousal and high valence levels. Figure 5 shows the biosensor responses for continuous emotion elicitation; 13 out of 14 OR activities happen exactly at the moments of the scenes eliciting the “joy” emotion (marked by vertical lines on the graph), only the first moment (marked as X in the upper graph) showing a false detection of OR. A validation on the “joy” emotional moments was further enhanced by the recording of the surrounding sound when viewing the film, and all related OR moments were accompanied by a subtle laughing sound of the subjects (see lower graph of Figure 5). Interestingly, there is no delay in the phasic OR synchronised with the joyful scenes similar to the still pictures experiment (see Section 4.1). However, it is likely to be a significant challenge to automatically identify the subject’s laughs from the complicated ambient sounds in real life.

## 5. Discussions

The present study has investigated three areas of emotion recognition based on physiological responses. Firstly, a proposal of a complete emotion recognition system emotionWear using affordable and readily available technologies was investigated that could synchronise between stimulation and the corresponding physiological responses. Our hypothesis is that synchronisation between system input and output can elucidate the relationship between emotional stimulation and the associated physiological variations. The collection of emotional pictures and film clips is easy to control in terms of stimulation timing. Synchronisation between stimuli and the corresponding responses is extremely helpful in analysing the effectiveness of a recognition method or device such as an individual biosensor. The framework emotionWear was designed and implemented from scratch to meet this purpose. The present research has used part of the functions of this emotion recognition analysing framework, which can be expanded: (1) to include all kinds of natural stimuli such as visual, auditory, haptic, olfactory and gustatory stimuli; (2) to capture the context during the study such as surrounding sounds and activities of the subjects; and (3) to monitor the physiological responses of the subject including all types of biomedical sensing data collected from available biosensors. The data analysis capability of emotionWear using jupyter notebook based mainly on Python has been proven to be a convenient tool for viewing both stimulation and emotional responses at the same time.

Based on the emotionWear framework, the second investigation focuses on the validation of a successful emotion elicitation, which enables a ground-truth database for emotion recognition using signal levels’ comparison or machine learning. The onset of OR activities is hypothesised to be an indicator for human attention and the initiation of the internal perception process [54,60]; thus, we want to illustrate this OR activity during emotion perception from emotionWear. A successful emotion elicitation is critical for enabling an accurate recognition especially in statistical prediction methods during both the training/testing and application phases. OR activities are hypothesised as the registry of sensory intake preparing the human (and animal) body to react to the changing environment due to novel stimuli. Numerous empirical studies have proven that the concomitant biological indicators (e.g., cardiac deceleration, skin conductance changes, pupil dilation) are associated with the initiation of the OR process. HR deceleration and rapid onset of EDA are being monitored in our framework validating a successful elicitation of emotions, and the results of our practical experiments agree with the hypothesis that the OR process is initiated when the stimuli (i.e., novel pictures and film clips that are significant to the participants) are applied to the subjects. The OR activities based on HR and EDA signals enable our framework to capture the emotional responses after the perception. However, according to the hypothesis, defensive response will be perceived during aversive stimuli, which causes HR acceleration instead. Therefore, different algorithms must be implemented in validating the emotion elicitation process, especially for humans with specific phobias.

The final investigation considers the coverage of emotion recognition using common off-the-shelf biosensors. Many previous empirical research works have hypothesised and proven to get high recognition accuracy using the three biosensors similar to the current study. We believe that each biosensor may have their individual irreplaceable feature in the recognition process. The OR process is indicated by sudden variations of skin conductance when novel stimulations are applied, and still pictures, short film clips and the longer film with multiple stimulations can verify this behaviour. However, a non-specific response happens at other moments not related to any specific stimulations, and this is a normal physiological response for humans [6]. EDA variations are concomitant to emotional arousal; thus, it is a common variable for autonomic specificity. Similar to EDA, HR deceleration is also an OR indicator illustrating the onset of the perception process. Again, the two branches of ANS are continuously innervating the cardiac acceleration and deceleration to support other physiological activities balancing the human biological systems. Therefore, the monitoring of both EDA and HR variation simultaneously gains a better estimation of OR activities. According to ANS specificity research, fingertip temperature helps to differentiate pleasant and unpleasant emotions, and our result has also demonstrated this response (see Table 5). The current study has also illustrated the possibility of emotion recognition with the level comparison method using only these three biosensors (i.e., PPT, EDA and SKT). The basic assumption is that a set of biosignals can be collected from a specific interval after a successful emotion elicitation process.

Theoretical cognitive research in attention and emotion is still in its infancy, and there are many questions waiting to be answered in the whole attentional processing of the environmental emotional stimuli [76]. OR is the next step after a selective attentional process, which is a highly complicated mechanism under extensive research and is showing extremely different attentional effects according to individuals with various physical and mental conditions [77,78]. However, the identification of the OR processes enables a way to capture the necessary environmental context of stimuli ready for the corresponding scientific methods to extract the primal components of stimulation.

Further expansion of this work is required to gain better understanding of the relationship between OR activities and emotion elicitations. The timing of cardiac deceleration, the duration of deceleration, the possible cardiac acceleration for subject-dependent phobia response and the spatial relationship between HR and EDA are critical variables for emotion recognition based on physiological responses. More data should be collected from different subjects on single emotional type, as well as multiple emotions in order to build a better statistical model for prediction.

## 6. Conclusions

An emotion prediction framework emotionWear has been proposed in this paper for predicting human emotions based on common wearable biosensors. The framework’s specific feature of synchronisation between emotional stimuli and the corresponding physiological responses enhances the data analysis of the coverage of emotional states from individual to a combination of biosensors. A novel algorithm based on the detection of the OR hypothesis has demonstrated the ability to validate a successful emotion elicitation through this synchronisation process. After a validation of successful emotion elicitation, the HR, EDA and SKT variations matched the ANS specificity by the accuracy confidence of HR: *p* < 0.126 (87.4%), EDA: *p* < 0.042 (95.8%) and SKT: *p* < 0.036 (96.4%).

Emotion has been researched for more than a century for humans and animals. Knowledge about emotion, especially the functions of emotion, has been heavily studied by researchers clarifying the mystery in many areas. There are still numerous related questions waiting to be answered from academia and industry, but the current understanding of emotion should be able to transfer the know-how into commercial applications such as AI and IoT. Biomedical engineering plays a key role in translating psychophysiological research results into practice through technology and provides dedicated and advanced tools such as accurate emotion recognition and prediction. The current research has established a strong background for emotion prediction through an experimental framework that collects a dataset of physiological response patterns synchronising with the associated stimuli under a valid emotion elicitation. Individual response specificity is also illustrated in the current framework, which shows that different subjects may behave differently, and even if their emotional behaviours are similar, the moments of emotional perception may be quite different. The RSS method to detect the OR activities was demonstrated as a validation tool for successful emotion elicitation, and this detection of the perception moments leading to an emotional response is extremely helpful in IoT environments where the implementation of affective sensors is enabled. Once the physiological responses are verified as coming from a true emotional perception, a combination of simple biosignal levels can be used with reasonable accuracy for emotion recognition by comparing them with the ANS specificity patterns. Machine learning can further increase the accuracy by extracting more features from various biosensors. More research is needed in clarifying the different OR activities for common discrete emotions, since it is necessary to accurately validate the emotional perception moments. A future study on the time delay after a successful emotion elicitation and the optimum period for collecting physiological signals is also important to increase the emotion recognition accuracy.

## Figures and Tables

**Table 1:** Feature extraction from PPG, EDA and SKT sensors.

PPG Features	Calculations Based on Python (Import Numpy, Pandas and Scipy)
IBI	Peak detection of raw PPG signal and get an array (ppgnn)
	Interbeat interval (IBI) = ppgnn.interpolate(method = ”cubic”)
HR	Heart Rate = (60 s × sampling frequency)/peak-to-peak duration
	HR = IBI.rolling (window, min_periods = 1, centre = True).mean()
SDNN	Standard deviation of IBI
	SDNN = IBI.rolling (window, min_periods = 1, centre = True).std()
SDSD	Standard deviation of the difference between adjacent ppgnn
	ppgdiff = pd.DataFrame(np.abs(np.ediff1d(ppgnn)))
	ppgdiff = ppgdiff.interpolate (method = “cubic”)
	SDSD = ppgdiff.rolling (window, min_periods = 1, centre = True).std()
RMSSD	Root Mean Square of the difference between adjacent ppgnn
	ppgsqdiff = pd.DataFrame (np.power(np.ediff1d (ppgnn), 2))
	ppgsqdiff = ppgsqdiff.interpolate (method = ”cubic”)
	RMSSD = np.sqrt (ppgsqdiff.rolling (window, min_periods = 1, centre = True).mean())
SDNN/RMSSD	Ratio between SDNN and RMSSD
	SDNN_RMSSD = SDNN / RMSSD
LF	Power Spectral Density (PSD) for low frequency range (0.04 Hz to 0.15 Hz)
	Y = np.fft.fft (IBI)/window, Y = Y [range(window//2)]
	LF = np.trapz (np.abs(Y[(freq ≥ 0.04) & (freq ≤ 0.15)]))
HF	PSD for high frequency range (0.16 Hz to 0.4 Hz)
	HF = np.trapz(np.abs (Y[(freq ≥ 0.15) & (freq ≤ 0.4)]))
LF/HF	PSD ratio between LF and HF
	LHF = LF / HF
**EDA Features**	**Calculations Based on Python (Import Numpy, Pandas and Scipy)**
EDA (filtered)	eda = raw EDA signal sampling at 100 ms
	B, A = signal.butter (2, 0.005, output = “ba”)
	EDAf = signal.filtfilt (B, A, eda)
EDA (mean)	Getting rolling mean of filtered EDA raw signal (EDAf)
	EDAmean = EDAf.rolling (window, min_periods = 1, centre = True).mean()
EDA (std)	Getting rolling standard deviation of filtered EDA raw signal (EDAf)
	EDAstd = EDAf.rolling (window, min_periods = 1, centre = True).std()
**SKT Features**	**Calculations Based on Python (Import Numpy, Pandas and Scipy)**
SKT (filtered)	skt = raw SKT signal sampling at 100 ms
	B, A = signal.butter (2, 0.005, output = “ba”)
	SKTf = signal.filtfilt (B, A, skt)
SKT (mean)	Getting rolling mean of filtered SKT raw signal (SKTf)
	SKTmean = SKTf.rolling (window, min_periods = 1, centre = True).mean()
SKT (std)	Getting rolling standard deviation of filtered SKT raw signal (SKTf)
	SKTstd = SKTf.rolling (window, min_periods = 1, centre = True).std()

**Table 2:** Elicitation of basic emotions using referenced stimuli.

Emotions	Pictures (Numbers Refer to IAPS Database [71])	Film Clips (Names and Duration of Film Clips Refer to Schaefer et al. [73])
Happiness/Joy	High valence rating:	(1) Something About Mary [2]
	#1710 (Puppies), #1750 (Bunnies),	(2) A fish called Wanda
	#5833 (Beach), #1460 (Kitten),	(3) When Harry met Sally
	#2050 (Baby), #1440 (Seal),	
Anger	#2040 (Baby), #2070 (Baby),	(1) Schindler’s list [2]
	#8190 (Skier), #2080 (Babies)	(2) Sleepers
		(3) Leaving Las Vegas
		
Fear	Low valence rating:	(1) The Blair Witch Project
	#3053 (BurnVictim), #3102 (BurnVictim),	(2) The Shining
	#3000 (Mutilation), #3064 (Mutilation),	(3) Misery
	#3170 (BabyTumor), #3080 (Mutilation),	
Disgust	#3063 (Mutilation), #9410 (Soldier),	(1) Trainspotting [2]
	#3131 (Mutilation), #3015 (Accident)	(2) Seven [3]
		(3) Hellraiser
		
Sadness		(1) City of angels
		(2) Dangerous mind
		(3) Philadelphia

**Table 3:** Film clips’ elicitation analysis.

Film Clips Target Emotions (And Distribution)	Emotion (Target = Subjective)	Emotion Elicitation (Subjective)	Emotion Elicitation (Measure)	Hit-Rate (Measure = Subjective)	Subjective Arousal (Average)	Subjective Valence (Average)
Joy (19%)	100%	19%	12%	60%	3.50	3.75
Anger (23%)	0%	0%	4%	0%	0.00	0.00
Fear (15%)	43%	27%	12%	43%	5.43	7.43
Disgust (21%)	71%	26%	19%	57%	5.57	8.14
Sadness (23%)	57%	18%	7%	43%	4.20	7.14

**Table 4:** Reasons for unsuccessful emotion elicitation.

Reasons for Unsuccessful Emotion Elicitation	Percentage
Saw the film clip before (many times)	17%
Film clip too short	42%
Cannot understand the language	4%
Did not feel the target emotion	33%
Others	4%

**Table 5:** Emotional recognition before and after validation of emotion elicitation.

Biosignal	Responses Match with ANS Specificity Before Successful Emotion Elicitation	Responses Match with ANS Specificity After Successful Emotion Elicitation
HR	*p* < 0.992	*p* < 0.126
EDA	*p* < 0.989	*p* < 0.042
SKT	*p* < 0.362	*p* < 0.036

## Appendix A. Off-The-Shelf Biomedical Sensors

Only common biomedical sensors that can be purchased from online resources are used in the present research, and their details are listed below.

- PPG (Photoplethysmography)
  Pulse Sensor is an open source hardware project by Joel Murphy and Yury Gitman, and the specification can be obtained from https://pulsesensor.com/pages/open-hardware.
- EDA (Electrodermal Activity)
  Grove GSR Sensor from Seeed; specification can be obtained from http://wiki.seeed.cc/Grove-GSR_Sensor/.
- SKT (Skin Temperature)
  Si7051 Digital Temperature Sensors with I2C Interface from Silicon Labs; specification can be obtained from https://www.silabs.com/products/sensors/si705x-temperature-sensors.
- EMG (Surface Electromyography)
  MyoWare Muscle Sensor from Advencer Technologies; specification can be obtained from http://www.advancertechnologies.com/p/myoware.html.
